# Discovery of a Potential HER2 Inhibitor from Natural Products for the Treatment of HER2-Positive Breast Cancer

**DOI:** 10.3390/ijms17071055

**Published:** 2016-07-01

**Authors:** Jianzong Li, Haiyang Wang, Junjie Li, Jinku Bao, Chuanfang Wu

**Affiliations:** 1 School of Life Sciences and Key Laboratory of Ministry of Education for Bio-Resources and Bio-Environment, Sichuan University, Chengdu 610064, China; lijianzong@hotmail.com (J.L.); haiyangwang1@outlook.com (H.W.); jimzz@alumni.sjtu.edu.cn (J.L.); 2 State Key Laboratory of Biotherapy/Collaborative Innovation Center for Biotherapy, West China Hospital, Sichuan University, Chengdu 610041, China; 3 State Key Laboratory of Oral Diseases, West China College of Stomatology, Sichuan University, Chengdu 610041, China

**Keywords:** HER2 (human epidermal growth factor receptor 2), virtual screening, ADMET (absorption, distribution, metabolism, excretion and toxicity), biological evaluation

## Abstract

Breast cancer is one of the most lethal types of cancer in women worldwide due to the late stage detection and resistance to traditional chemotherapy. The human epidermal growth factor receptor 2 (HER2) is considered as a validated target in breast cancer therapy. Even though a substantial effort has been made to develop HER2 inhibitors, only lapatinib has been approved by the U.S. Food and Drug Administration (FDA). Side effects were observed in a majority of the patients within one year of treatment initiation. Here, we took advantage of bioinformatics tools to identify novel effective HER2 inhibitors. The structure-based virtual screening combined with ADMET (absorption, distribution, metabolism, excretion and toxicity) prediction was explored. In total, 11,247 natural compounds were screened. The top hits were evaluated by an in vitro HER2 kinase inhibition assay. The cell proliferation inhibition effect of identified inhibitors was evaluated in HER2-overexpressing SKBR3 and BT474 cell lines. We found that ZINC15122021 showed favorable ADMET properties and attained high binding affinity against HER2. Moreover, ZINC15122021 showed high kinase inhibition activity against HER2 and presented outstanding cell proliferation inhibition activity against both SKBR3 and BT474 cell lines. Results reveal that ZINC15122021 can be a potential HER2 inhibitor.

## 1. Introduction

The World Health Organization (WHO) reports that breast cancer is one of the most common malignancies among women worldwide. Breast cancer has a substantially higher incidence (43.3 per 100,000) than any other cancers, followed by colorectal (14.3), cervix (14.0), lung (13.6) and corpus uteri (8.2) among gynecological tumors [1]. Despite a relatively low fatality rate, breast cancer has the highest death rate (12.9 per 100,000) than any other cancers. Although molecular therapies for breast cancer have developed rapidly over the last few decades [2], future treatment strategies with higher efficacy and lower toxicity are still in urgent need to be investigated.

The human epidermal growth factor receptor 2 (HER2) is a validated target in breast cancer therapy. HER2, a transmembrane receptor with tyrosine kinase activity, belongs to the epidermal growth factor receptor family [3] and is known as a potent mediator of cellular growth and proliferation. It plays a significant role in many processes implicated in the regulation of cell survival, growth and differentiation through interlinked signal transduction involving activation of the PI3K/Akt and MAPK/ERK1/2 (mitogen-activated protein (MAP) kinase/extracellular signal-regulated kinase (ERK)1/2) pathways [4,5]. Some studies demonstrated that HER2 is always in an active conformation and ready to interact with the ligand-activated HER receptors [6]. Overexpression of HER2 is one of the molecular abnormalities linked to the development of breast cancer [7] and exists in about 30% of patients with early stage breast cancer [8,9].

In the era of molecular and personalized therapeutics, the discovery of tyrosine kinase inhibitors targeting HER2 has provided a successful avenue of therapies in HER2-overexpressing breast cancer [10]. Many small molecules inhibitors targeting HER2 are currently in clinical development [11]. Lapatinib, an orally active HER2 kinase inhibitor, is the only drug approved by the U.S. Food and Drug Administration (FDA) for patients with HER2-positive advanced-stage breast cancer specifically [12]. It competes with ATP to bind with HER2 kinase domain, suppressing HER2 kinase activity and followed by shutting off downstream pathways [13]. Clinical trials demonstrated that lapatinib could enhance the apoptotic effect of anti-HER2 antibodies [14,15], and a combination of lapatinib and monoclonal antibody had better efficacy than lapatinib alone [16,17]. However, it can be effective initially, and then the patients with HER2-positive breast cancer develop acquired drug-resistance with one year of treatment [17,18]. There are two synthetic compounds as HER2 inhibitors that are currently in Phase III trials. These drug candidates, including dacomitinib (PF-00299804, Pfizer, New York, NY, USA) [19,20] and neratinib (Pfizer) [21], are relatively similar to lapatinib. Although tremendous progress in HER2-directed therapies has been made, a significant proportion of HER2-positive patients still relapse and die of breast cancer. Hence, alternative therapies are urgently required. 

**Figure 1:**
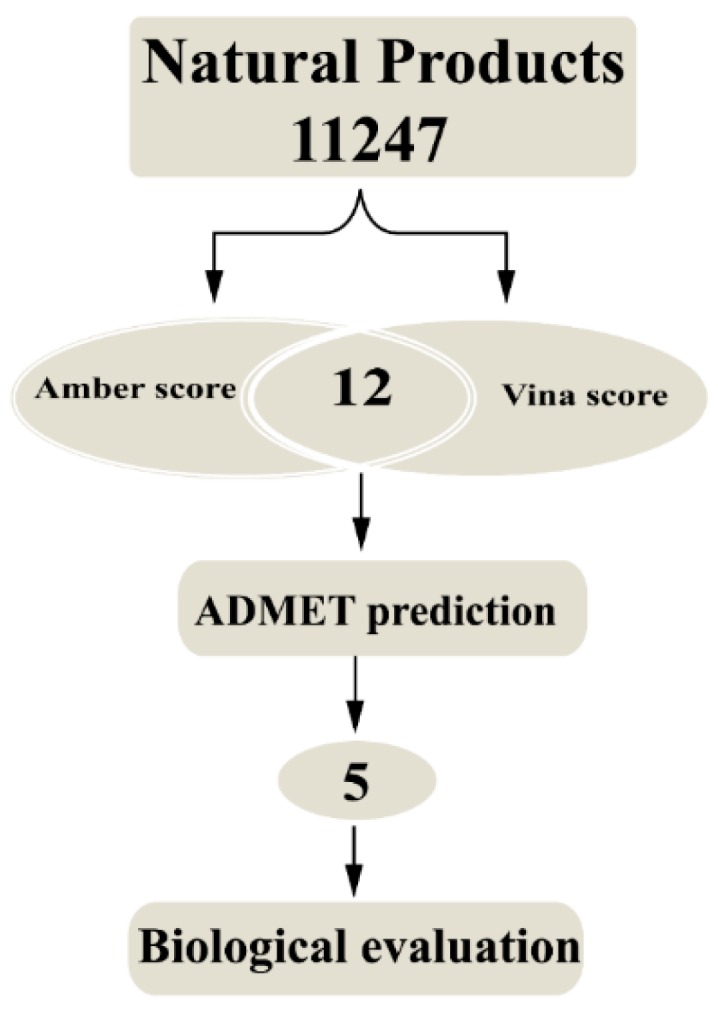
The brief workflow of identification of novel potential inhibitors targeting human epidermal growth factor receptor 2 (HER2).

For the purpose of developing novel potential HER2 inhibitors, the structure-based high-throughput virtual screening has been performed. The ADMET (absorption, distribution, metabolism, excretion and toxicity) properties of small molecule candidates were also evaluated. Moreover, the MM–PBSA (Molecular Mechanics–Poisson Boltzmann Surface Area) calculation based on molecular dynamics (MD) simulation was utilized to further investigate the binding activity of the HER2-inhibitor systems. Furthermore, the selected inhibitors were evaluated by an in vitro HER2 kinase activity inhibition assay. The cell proliferation inhibition was tested in HER2-overexpressing SKBR3 and BT474 cell lines. The brief workflow was presented in Figure 1. The potential HER2 inhibitors identified from the current study could be helpful in the design and development of a novel HER2 inhibitor.

## 2. Results

### 2.1. Molecular Docking Study

Here, Amber scores and Autodock Vina scores were calculated in parallel with the 11,247 natural compounds. Twelve common compounds showing both high Amber scores and high Vina scores were selected. Lapatinib and the natural substrate ATP were also docked into HER2. The results were presented in Table 1. Compared to lapatinib, seven compounds showed higher Amber score, six compounds showed higher Vina score, four compounds showed both higher Amber and Vina scores. All compounds showed both higher Amber scores and Vina scores than the natural substrate ATP. Meanwhile, we found a common structural characteristic for selected compounds that hydrogen-bonding groups and nonpolar aromatic moieties were situated in the middle and at the end of molecular frameworks, respectively.

**Figure 2:**
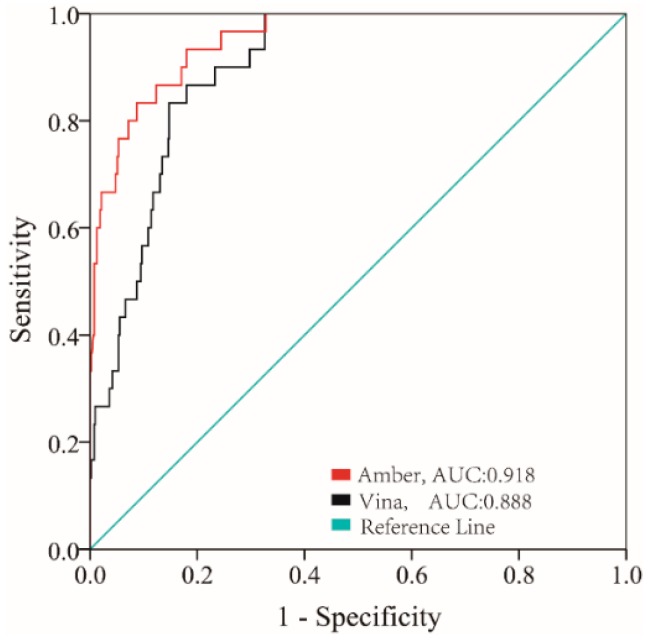
Receiver operating characteristic (ROC) analysis of the simulated docking model.

In order to ensure the reliability of the screening model for high throughput virtual screening, an evaluation was performed. The output of the receiver operating characteristic (ROC) is shown in Figure 2. The Amber score achieved a AUC (the area under the ROC curve) of 0.918, the Vina score achieved an AUC of 0.888. The results indicated that the molecular docking model was reliable for distinguishing the active compounds and inactive compounds effectively.

### 2.2. ADMET (Absorption, Distribution, Metabolism, Excretion and Toxicity) Properties Analysis

Favorable ADMET characteristics can be considered as an essential nature for a candidate drug. The ADMET properties of 12 selected natural compounds were predicted. Based on ADMET prediction, five potential compounds showed favorable ADMET properties. The selected prediction properties of the top five hits are shown in Table 2. Rule-of-five (RO5) represents physicochemical parameters defined by Lipinski [22], which follows: (1) molecular weight lower than 500; (2) number of H-bond (hydrogen bond) donors lower or equal to five; (3) number of H-bond acceptors lower or equal than 10; and (4) log *P* lower than five. None of the selected compounds violated this rule. ADMET Risk indicates the comprehensive evaluation of ADMET properties. All compounds attained this value lower or equal to five. The results demonstrated that these compounds possessed outstanding ADMET properties.

### 2.3. Molecular Simulation Analysis

**Figure 3:**
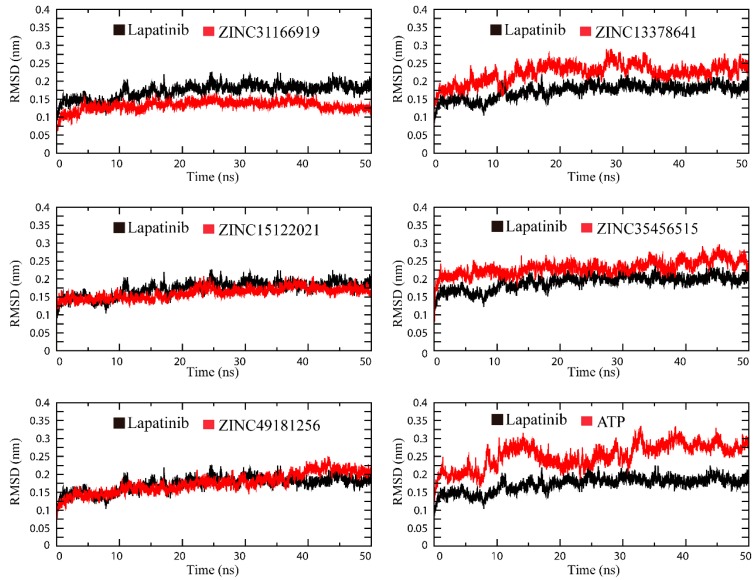
The RMSD (root mean square deviations) of the backbone atoms of HER2-ligand systems.

Assessment of RMSD (root mean square deviations) value for each HER2-ligand system provided a complete insight into the conformational stability of each complex system. The RMSD values of five selected compounds, lapatinib and ATP were shown in Figure 3. As can be seen, most of the selected compounds possessed high stability thoughout the 50 ns MD simulation. In particular, ZINC31166919 (red) and ZINC15122021 (red) showed minimum fluctuation and attained an average RMSD value at 0.12 nm and 0.14 nm during 50 ns MD simulation, respectively. ZINC49181256 (red) also showed low fluctuation and attained an average RMSD value about 0.150 nm. By contrast, lapatinib (black) attained an average RMSD value about 0.17 nm. It demonstrated that ZINC31166919, ZINC15122021 and ZINC49181256 could bind to HER2 very stably and more tightly than lapatinib. The fluctuation of RMSD of ZINC3545651 and ZINC13378641 was slightly higher than other compounds. Although the fluctuation of RMSD indicated that ZINC3545651- and ZINC13378641-HER2 systems were not stable as others, the maximum RMSD values of all selected compounds were lower than 0.3 nm. The natural substrate ATP showed higher RMSD value than other compounds and the fluctuation of RMSD over time indicated that ATP did not possess much unfavorable stability against HER2 as compared to other compounds.

### 2.4. Binding Affinity Prediction

The MM–PBSA tool was employed to calculate their binding free energy. Generally, the ∆*Gbind* indicates the comprehensive evaluation of binding affinity. The detailed results were presented in Table 3. It is encouraging to observe that two compounds ZINC31166919 (−131.36 kcal/mol) and ZINC15122021 (−120.63 kcal/mol) showed more favorable binding affinity compared to other compounds as well as lapatinib (−37.49 kcal/mol). Three compounds showed better binding effect with HER2 than lapatinib, including ZINC15122021, ZINC31166919 and ZINC49181256. In contrast, two compounds, including ZINC13378641 and ZINC35456515, showed slighter unfavorable binding affinity than lapatinib. The results were consistent with the trajectory analysis as lower binding energy indicated more favorable binding stability.

### 2.5. Biological Evaluation

**Figure 4:**
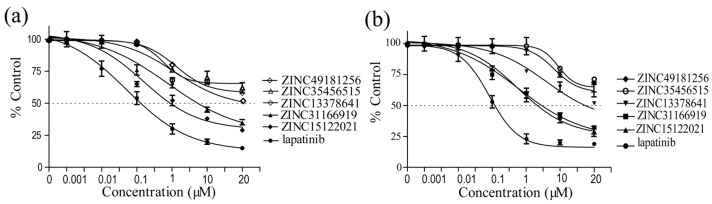
Dose-response effect of selected compounds on breast cancer cell lines, (**a**) SKBR3 cell line; (**b**) BT474 cell line. Results are expressed as the mean percentage of control plates containing no drug. Error bars correspond to SD (standard deviations) from three independent measurements.

For the purpose of seeking novel potential HER2 inhibitors, HER2 inhibition assay and cell proliferation inhibition were evaluated in vitro. Most of the selected natural compounds showed a moderated kinase inhibition activity against HER2, and only a few showed outstanding inhibition activity. The cellular inhibition activities were mainly coincident with the results of kinase inhibition. The data were detailed in Table 4, and the cell growth curves were presented in Figure 4.

In particular, we found that ZINC15122021 showed high inhibition activity against HER2 and presented favorable cell proliferation inhibition activity against both SKBR3 and BT747 cell lines. It is encouraging find that ZINC15122021 exhibited high activities with IC_50_ value of 0.18 μM against HER2, IC_50_ value of 1.22 μM against SKBR3 cells and 4.11 μM against BT474 cells. ZINC31166919 also exhibited high activities with IC_50_ value of 2.63 μM against HER2, IC_50_ value of 8.61 μM against SKBR3 and 6.78 μM against BT474. ZINC13378641 showed lower activities than these compounds. Even though their activities were slightly lower compared with lapatinib, their cell inhibition IC_50_ values were lower than 50 μM except ZINC49181256 and ZINC35456515.

**Figure 5:**
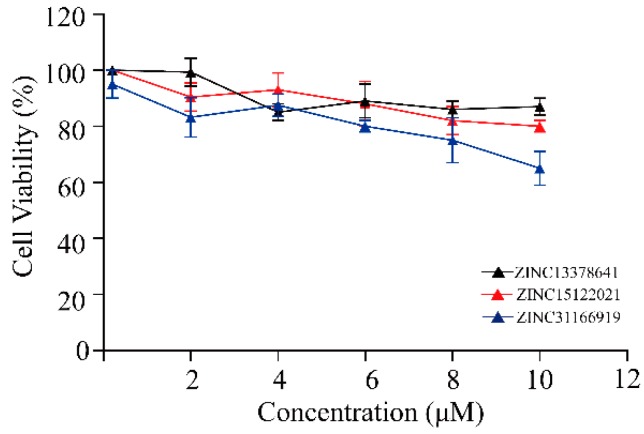
Cell viability of normal breast cells treated with three potential HER2 inhibitors by using CCK-8 assay. Error bars correspond to standard deviations from three independent measurements.

Three compounds (cell inhibition IC_50_ values < 50 μM) were further tested on the normal breast cell (Hs578Bst) to study the effect on cell proliferation and thus verify the toxicity of normal cells. CCK-8 (Cell Counting kit-8) assays were utilized to determine the effects of different concentrations of three compounds on normal breast cell viability after 24 h of treatment. Our experiment results revealed that ZINC15122021 and ZINC13378641 exhibited little cytotoxicity against normal breast cells. ZINC31166919 showed slightly enhanced cytotoxicity with increase of concentration (see Figure 5).

### 2.6. Binding Model Analysis

**Figure 6:**
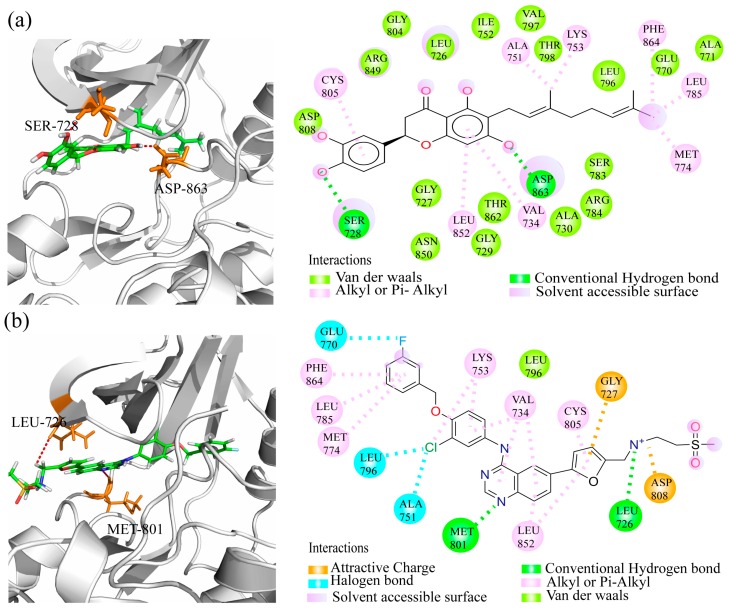
Comparison of binding models of lapatinib and ZINC15122021 against HER2 at atomic level. (**a**) the interaction of ZINC15122021 with HER2; (**b**) the interaction of lapatinib with HER2. The 2D diagram interactions were shown as dashed lines between receptor residues and ligand atoms. The figures were generated by the PyMOL1.5 (The PyMOL Molecular Graphics System, San Carlos, CA, USA) and Discovery Studio visualizer 16 (Accelrys, San Diego, CA, USA).

The results indicated that lapatinib could interact with Leu726 and Met801 residues, whereas ZINC15122021 could interact with Ser728 and Asp863 residues in the HER2 ATP-binding pocket. The conventional hydrogen bonds were obviously found in these residues, see Figure 6. According to the docking study, we found that van der Waals interactions played a key role between ZINC15122021 and HER2. Meanwhile, the Alkyl or Pi-Alkyl interactions were also obviously found between ligands and HER2. Compared to ZINC15122021, lapatinib formed halogen bonds with Ala751, Glu770 and Leu796 residues. In addition, two attractive or repulsive charged interactions were found between lapatinib and Gly727 and Asp808 residues. Various interaction types may be the reason that lapatinib can achieve high binding affinity and possess high HER2 kinase inhibition. In fact, van der Waals is one of the dominating forces for HER2-ZINC15122021 binding. It is the weakest of all intermolecular attractions between molecules, however, with lots of van der Waals force interactions between ligand and receptor, the interaction can be very strong. Thus, ZINC15122021 can also achieve high binding affinity with HER2 and possess high biological activities against HER2.

## 3. Discussion

According to the classical magic bullet paradigm, once biochemical and genetic studies reveal the molecular mechanisms of diseases such as cancer, it becomes possible that people could pick a protein that they think would make a favorable target, and screen compounds that interact with this protein as potential drugs. Even with its current limitations, computational virtual screening offers a practical pipeline to discover new potential drugs for pharmaceutical research [23,24]. Many novel ligands have been successfully discovered using structure-based computation [25,26,27,28]. The remarkable features that discriminate the present study from the other concerns are as follows:

(1) a large number of natural compounds are docked into the ATP-binding pocket of HER2. Studies have indicated that natural products play a highly significant role in the drug discovery and development process [29];

(2) the high AUC values indicates that our virtual screening model was reliable for identifying active ligands from compounds database effectively;

(3) one of the most daunting hurdles a drug candidate must pass is possessing suitable ADMET properties [30]. Identification of these properties during early drug discovery is vital for reducing ADMET problems later in the drug development process. In present study, the ADMET properties of several selected compounds were analyzed. Our results reveal that five natural compounds possessed favorable ADMET properties;

(4) meanwhile, these compounds were tested on the normal breast cell line to verify their toxicity on normal cells. We found that ZINC15122021 and ZINC31178641 possessed low cytotoxicity of normal breast cell. It indicates that these natural compounds can reduce the possibility of loss in drug discovery process;

(5) biological evaluation act as gatekeeper by assessing enzymatic activity assay and cancer cell proliferation inhibition. Based on virtual screening results, the biological activities of five compounds were further tested. Although inhibition activities of ZINC15122021 were slightly lower than lapatinib, we found ZINC15122021 possessed high biological activities against HER2, and exhibited the IC_50_ values of 0.18 μM against HER2, 1.22 μM against SKBR3 cell and 4.11 μM against BT474 cells, making it promising to be an effective HER2 inhibitor.

## 4. Methods

### 4.1. Receptor and Ligand Preparation

The crystal structure of HER2 kinase domain with high resolution used for the present study was retrieved from the RCSB protein databank: 3PP0 (X-ray diffraction with resolution 2.25 Å). The three-dimensional structures of all 11,247 natural products prepared for molecular docking were obtained from AnalytiCon Discovery NP [31]. The structure of lapatinib and natural substrate ATP were retrieved from the ZINC database.

### 4.2. Molecular Docking Model Validation

In order to identify molecular docking models suitable for virtual screening against HER2, we used the crystal structure of the HER2 kinase domain with high resolution to assess the performance of virtual screening model. Herein, a docking simulation test was explored and the ROC was further analyzed. Thirty compounds were considered as the positive dataset, which were collected from literatures and the ChEMBL database according to its affinities (IC_50_, *K*_i_ and *K*_d_ ≤ 100 nM). In addition, 561 decoys found by DecoyFinder1.1 [32]. The parameters of DecoyFinder1.1 were set as the following: active ligand vs. decoy tanimoto threshold ≤0.75; decoy vs. decoy tanimoto threshold <0.9; hydrogen bond donors ±1, hydrogen bond acceptors ±2; molecular weight ±25 Da; and rotational bonds ± 1, log *P* ± 1.00. The decoys were obtained from HER2 decoys in the DUD-E database [33]. The positive ligands and the decoys were used as input datasets for the docking simulation test model. The AUC values were calculated by the R package.

### 4.3. Molecular Docking

In order to investigate the binding effect of HER2 with ligands, the molecular docking was employed by the DOCK6.5 [34] and AutoDock Vina v1 program [35]. The Dock prep tool of UCSF Chimera [36] was used for protein preparation including energy minimized and water removal. The coordinates of structures were complexed with water molecules and other atoms responsible for increased resolution, thus the additional atoms were removed using Chimera. The Amber score, which enables all or part of the receptor to be flexible, was calculated by the DOCK6.5 program. The Amber score implements molecular mechanics, implicit solvent and molecular dynamic simulations based on the traditional all-atom. Protein and ligand were dealt with the general AMBER force field [37]. The Vina score was calculated by AutoDock Vina program. The detailed parameters refer to our previous studies [38,39,40,41].

### 4.4. ADMET Prediction

ADMET properties for all the selected ligands were predicted by ADMET Predictor 6.5 (Simulations Plus Inc., Lancaster, CA, USA) [42,43], and the ADMET properties can be utilized to estimate crucial physicochemical or biological attributes for large numbers of drug-like compounds. The ADMET predictor has been consistently ranked as the most effective tool to predict physicochemical and biological attributes of potential drug-like compounds. Its predictive protocols include physicochemical, biopharmaceutical, metabolism, toxicity and simulation modules.

### 4.5. MM/PBSA Binding Based on Molecular Dynamic Simulation Affinity Prediction

MD simulations were performed for a period of 50 ns by the Gromacs 5.0 (GROningen MAchine for Chemical Simulation) [44]. The system consisted of (1) the ligand-receptor complex, which was solved using TIP3P waters [45,46]; (2) Na^+^ and Cl^−^ ions neutralizing the system; and (3) periodic boundary conditions with a minimal distance of 1.0 between the protein and the edge of the box. An ff99SB force field [47] was used for the protein and GAFF (a general AMBER force field) parameters [48] for the ligand that comes from Amber Tools (San Francisco, CA, USA) [49]. At the beginning of MD simulations, the receptor topology files were converted by the pdb2gmx program. Then, the system was subjected to two phases of equilibration for a period of 1000 ps at constant temperature (300 K) along with constant pressure (1 atm), the two phases consisted of (1) constant number of particles, volume, and temperature (NVT) and (2) constant number of particles, pressure, and temperature (NPT). Following equilibration, MD with a time step of 2 fs were performed. The covalent bond lengths were constrained using the Linear Constraint Solver algorithm [36] and long-range electrostatic interactions were calculated using the Particle Mesh Ewald (PME) method [50]. The SETTLE algorithm [51] was used for the water molecules. The trajectory information was collected every 2 ps for further analysis. Finally, MD simulations were performed for a period of 50 ns.

The MM–PBSA were performed by *g_mmpbsa* tool [52,53]. In MM–PBSA, the binding free energy is calculated as follows:

Δ*G_bind_* = ∆*H* − *T*∆*S* ≈ ∆*G_sol_* + ∆*E_MM_* − *T*∆*S* (1)

∆*E_MM_* = ∆*E_internal_* + ∆*E_vdw_* + ∆*E_electrostatic_* (2)

∆*G_sol_* = ∆*G_SA_* + ∆*G_PB_* (3)

where ∆*G_sol_* is the changes of the solvation free energy, ∆*E_MM_* is the gas phase MM energy, −*TΔS* is the changes of the conformational entropy upon binding, ∆*E_internal_* is bond, angle and dihedral energies, ∆*E_electrostatic_* is electrostatic, ∆*E_vdw_* is van der Waals energies, and ∆*G_sol_* is the sum of the no electrostatic solvation component (nonpolar contribution, e.g., ∆*G_SA_*) and the electrostatic solvation energy (polar contribution, e.g., ∆*G_PB_*) [52].

### 4.6. In Vitro Enzymatic Activity Assay and Cell Proliferation Inhibition

The compounds were tested for HER2 kinase inhibition abilities using an HER2 assay kit (Invitrogen, Carlsbad, CA, USA, PV3366). The kinase kit was obtained from Invitrogen; all compounds were purchased from Thermo Electron Corporation (Walthan, MA, USA). The concentrations of HER2 were set as 0.20 μg/mL. The concentration of selected compounds was set as six gradients (0.0001–10 μM) in DMSO. Firstly, 2.5 μL of ATP solution and 2.5 μL of compound solution was added to the 5.0 μL substrate peptide. Then, the mixture solution on the plate was incubated for 1 h at 25 °C. Next, 5 μL of development solution was added to the well, and the plate was incubated for 1 h at 25 °C. To terminate the reaction, 5 μL of stop reagent was added to plate. The Infinite M100 Pro multi-label reader (Tecan, Männedorf, Switzerland) was used to measure the activity. Graph Pad Prism 5.0 (Graph Pad Software, San Diego, CA, USA) was used to calculate the IC_50_ values.

All cells were obtained from ATCC (Manassas, VA, USA). The HER2-overexpression SKBR3 cell line and BT474 cell line were cultured in RPMI (Roswell Park Memorial Institute medium) 1640 medium and supplemented with 10% FBS (fetal bovine serum). In addition, 100 U/mL of Penicillin and Streptomycin were added. The cell cultures were maintained at 37 °C in a humidified atmosphere of 5% CO_2_. Cells were seeded at density 2 × 10^4^/mL in 96-well plates per well. The medium was removed after seeding 24 h. The compounds were dissolved in DMSO (dimethyl sulfoxide) and diluted with medium to different concentrations. Then, 20 µL of the selected compounds solution were added in 96-well plates, and incubation continued for 48 h at 37 °C in a humidified atmosphere of 5% CO_2_. After that, the medium was removed, 90 µL of fresh medium and 10 μL of CCK-8 [54] (Dojindo Molecular Technologies Inc., Kumamoto, Japan) was added to each well and incubated for additional half hour. The activity was measured by microplate reader. Graph Pad Prism 5.0 was used to calculated the IC_50_ values. The normal breast cells (Hs578Bst) were cultured the same as SKBR3 cells. Cell viability was measured by the CCK-8 assay as previously described [55].

## 5. Conclusions

In summary, the present study serves as a preliminary study providing accurate information for the identification of novel HER2 inhibitors. The structure-based virtual screening combined with ADMET prediction, MD and MM–PBSA successfully identified several potential HER2 inhibitors. Then, in vitro kinase activity and cancer cell proliferation inhibition were performed to evaluate their biological activity. Overall, we identified the natural compound ZINC15122021 as a potential inhibitor against HER2. We believe that the identified natural compounds with outstanding ADMET properties and in vitro activities may guide the development of novel HER2 inhibitors.

## Figures and Tables

**Table 1:** The results of virtual screening and structures of common top-score compounds.

Rank ^a^	ZINC ID	Structure ^b^	Score (kcal/mol)	
			Amber Score	Vina Score
1	31166919	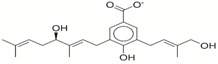	−44.19	−10.3
2	15122021	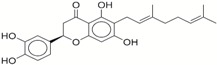	−43.67	−10.8
3	13378641	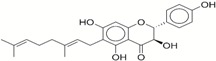	−43.54	−10.6
4	72320250	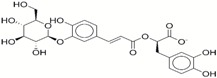	−35.54	−10.0
5	49181256	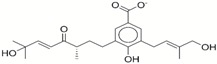	−35.26	−9.9
6	35456612	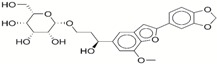	−34.01	−10.4
7	72320169	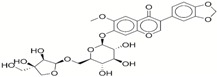	−33.93	−9.9
8	lapatinib	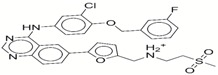	−32.59	−10.2
9	35456515	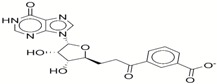	−33.55	−10.7
10	35456607	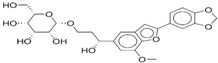	−32.45	−10.4
11	72320025	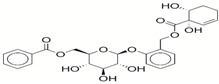	−32.01	−10.1
12	67912776	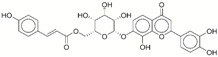	−31.85	−10.0
13	44352487	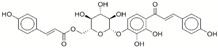	−28.83	−10.1
14	ATP	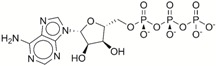	−10.45	−7.5

^a^ The compounds were sorted by Amber score; ^b^ The structures were generated by the ChemDraw program (CambridgeSoft, Cambridge, MA, USA). More information on these compounds was provided in Table S1.

**Table 2:** The ADMET (absorption, distribution, metabolism, excretion and toxicity) properties of the top five hits.

ADMET Properties	Molecules				
	ZINC13378641	ZINC15122021	ZINC35456515	ZINC31166919	ZINC49181256
S + logP	4.73	5.01	−0.01	3.92	2.92
S + Sw	1.10	5.97	1.08	1.00	1.11
S + Vd	0.7	0.46	0.26	0.17	0.13
CYP_1A2_Substr	No (66%)	No (59%)	No (96%)	No (96%)	No (96%)
MET_UGT1A1	No (59%)	Yes (58%)	No (92%)	No (88%)	No (82%)
TOX_hERG_Filter	No (95%)	No (95%)	No (95%)	No (95%)	No (95%)
TOX_BRM_Rat	289.81	522.34	9.89	200.29	206.47
TOX_AlkPhos	Normal (60%)	Normal (74%)	Elevated (65%)	Elevated (97%)	Elevated (97%)
TOX_GGT	Normal (78%)	Normal (78%)	Normal (57%)	Elevated (77%)	Elevated (90%)
TOX_LDH	Normal (76%)	Normal (76%)	Elevated (75%)	Normal (70%)	Normal (96%)
RO5	0	0	0	0	0
TOX_MUT_Risk	0	0	0	0	0
ADMET Risk	3.36	3.81	4.5	5	5

Where S + logP, S + Sw mean the octanol-water partition coefficient and native water solubility; S + Vda is the pharmacokinetic volume of distribution in human; CYP_1A2_Substr is the measurement of compound being the substrate of Cytochrome P450 1A2; MET_UGT1A1 is qualitative model of a glucuronidation by the UDP-glucuronosyltransferase 1A1 enzyme; TOX_hERG_Filter and TOX_AlkPhos denote qualitative estimation of the likelihood of the hERG potassium channel inhibition and liver adverse effect as the likelihood of causing elevation in the levels of Alkaline Phosphatase enzyme in human; TOX_BRM_Rat means the oral dose of compound required to cause tumors 50 percent of a rat population after exposure over an average lifetime; TOX_GGT means human liver adverse effect as the likelihood of causing elevation in the levels of GGT enzyme; TOX_MUT_Risk indicates ADMET Risk for mutagenicity in *S. typhimurium*.

**Table 3:** Summary of the binding free energy components for the protein–ligand complexes calculated by MM–PBSA (Molecular Mechanics–Poisson Boltzmann Surface Area) method.

Components	Molecules					
	ZINC31166919	ZINC15122021	ZINC49181256	ZINC13378641	ZINC35456515	Lapatinib
∆*Evdw*	−56.57 ± 1.95	−63.46 ± 2.58	−53.88 ± 0.82	−51.56 ± 2.84	−57.82 ± 3.09	−51.02 ± 3.39
∆*Eele*	−130.90 ± 7.19	−109.18 ± 6.70	−39.73 ± 0.77	−1.58 ± 5.65	−17.31 ± 10.65	−26.03 ± 8.63
∆*Gpb*	61.47 ± 5.13	57.30 ± 3.87	44.88 ± 1.18	24.14 ± 5.35	49.84 ± 7.13	45.25 ± 5.22
∆*Gsa*	−5.36 ± 0.19	−5.30 ± 0.21	−5.74 ± 0.06	−5.37 ± 0.18	−5.76 ± 0.21	−5.69 ± 0.21
∆*Emm*	−187.47 ± 4.57	−172.63 ± 4.64	−93.61 ± 1.59	−49.99 ± 4.24	−75.84 ± 6.87	−77.05 ± 6.01
∆*G_sol_*	56.11 ± 5.32	52.00 ± 3.66	39.04 ± 1.12	18.77 ± 5.17	44.08 ± 6.92	39.56 ± 5.01
∆*Gbind*	−131.36 ± 6.63	−120.63 ± 5.18	−54.44 ± 0.84	−31.22 ± 3.89	−31.05 ± 6.23	−37.49 ± 5.46

**Table 4:** The IC_50_ (half maximal inhibitory concentration) values of selected natural compounds against HER2 kinase and HER2-overexpressing SKBR3 and BT474 cell lines. The values represented the mean ± SD (standard deviations) of at least three independent experiments.

Compounds	Enzymatic IC_50_	Cell Inhibition IC_50_	
		SKBR3	BT474
ZINC31166919	2.63 ± 0.03	8.61 ± 0.45	6.78 ± 0.68
ZINC15122021	0.18 ± 0.002	1.22 ± 0.05	4.11 ± 0.95
ZINC49181256	9.18 ± 0.01	>50	>50
ZINC13378641	3.71 ± 0.03	26.48 ± 1.62	18.55 ± 2.06
ZINC35456515	>10	>50	>50
lapatinib	0.06 ± 0.001	0.38 ± 0.02	0.45 ± 0.03

## Supplementary Materials

Supplementary materials can be found at http://www.mdpi.com/1422-0067/17/7/1055/s1.
